# Nanostructural Modulation of G‐Quadruplex DNA in Neurodegeneration: Orotate Interaction Revealed Through Experimental and Computational Approaches

**DOI:** 10.1111/jnc.16296

**Published:** 2025-01-20

**Authors:** Andrea Patrizia Falanga, Ilaria Piccialli, Francesca Greco, Stefano D'Errico, Maria Grazia Nolli, Nicola Borbone, Giorgia Oliviero, Giovanni N. Roviello

**Affiliations:** ^1^ Department of Pharmacy University of Naples Federico II Naples Italy; ^2^ Division of Pharmacology, Department of Neuroscience, Reproductive and Dentistry Sciences, School of Medicine University of Naples Federico II Naples Italy; ^3^ ISBE‐IT, University of Naples Federico II Naples Italy; ^4^ Department of Molecular Medicine and Medical Biotechnologies University of Naples Federico II Naples Italy; ^5^ Institute of Biostructures and Bioimaging Italian National Council for Research (IBB‐CNR) Naples Italy

**Keywords:** amyotrophic lateral sclerosis (ALS), food drugs, frontotemporal dementia (FTD), G‐quadruplex DNA, orotate, orotic acid

## Abstract

The natural compound orotic acid and its anionic form, orotate, play a pivotal role in various biological processes, serving as essential intermediates in pyrimidine *de novo* synthesis, with demonstrated connections to dietary, supplement, and neurodrug applications. A novel perspective on biomolecular aggregation at the nanoscale, particularly pertinent to neurodegeneration, challenges the established paradigm positing that peptide (amyloid beta) and protein (tau) aggregation mainly govern the molecular events underlying prevalent neuropathologies. Emerging biological evidence indicates a notable role for G‐quadruplex (G4) DNA aggregation in neurodegenerative processes affecting neuronal cells, particularly in the presence of extended (G_4_C_2_)_n_ repeats in nuclear DNA sequences. Our study concerns d[(GGGGCC)_3_GGGG], a G4‐forming DNA model featuring G_4_C_2_ repeats that is in correlation with neurodegeneration. Through different investigations utilizing spectroscopic techniques (CD, UV, and thermal denaturations), PAGE electrophoresis, and molecular docking, the study explores the influence of orotate on the aggregation of this neurodegeneration‐associated DNA. A computational approach was employed to construct an *in silico* model of the DNA aggregate, which involved the docking of multiple G4 units and subsequent integration of the ligand into both the DNA monomer and its *in silico* aggregated model. The convergence of computational analyses and empirical data collectively supports the hypothesis that orotate possesses the capability to modulate the aggregation of neurodegeneration‐related DNA. Notably, the findings suggest the potential utility of orotate as a neurodrug, especially for the therapy of amyotrophic lateral sclerosis (ALS) and Frontotemporal Dementia (FTD), with its current status as a dietary supplement indicating minimal safety concerns. Additionally, orotate demonstrated a slight increase in mitochondrial dehydrogenase activity as assessed by the MTT assay, which is beneficial for a neurodrug as it suggests a potential role in enhancing mitochondrial function and supporting neuronal health.

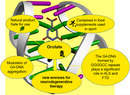

## Graphical abstract

Aim of this study is to investigate drugs that modulate GGGGCC repeat aggregation, a key feature in neurodegenerative diseases such as ALS and FTD. The research emphasizes the role of the *C9orf72* gene, which is implicated in these disorders, and focuses on G‐quadruplex (G4) DNA structures formed by these repeats. Orotate emerges as a promising modulator of G4 stability, influencing its formation and stability. These findings offer valuable insights into the potential of orotate for therapeutic interventions targeting G4 structures in neurodegeneration.

**Abbreviations**
3D: three dimensional
AD: alzheimer disease
ALS: amyotrophic lateral sclerosis
BD: bipolar disorder
CD: circular dichroism
CH_3_CN: acetonitrile
CHARMm: chemistry at Harvard macromolecular mechanics
CO_2_
: carbon dioxide
CPG: controlled pore glass
CTL: control
DHO: dihydroorotate
DHODH: dihydroorotate dehydrogenase
DMEM: Dulbecco's modified Eagle's medium
DMSO: dimethylsulfoxide
DNA: deoxyribonucleic acid
DS: discovery studio
EDTA: ethylenediaminetetraacetic acid
ESI‐MS: electrospray ionization mass spectrometry
FTD: frontotemporal dementia
G4: G‐quadruplex
HPLC: high‐performance liquid chromatography
KCl: potassium chloride
KH_2_PO_4_
: potassium dihydrogenphosphate
Li_2_CO_3_
: lithium carbonate
LiOr: lithium orotate
MTT: 3‐[4,5‐dimethylthiazol‐2‐yl]‐2,5‐diphenyl tetrazolium bromide
NaCl: sodium chloride
NaH_2_PO_4_
: sodium dihydrogenphosphate
NMR: nuclear magnetic resonance
OA: orotic acid
ODN: oligodeoxyribonucleotide
OPRT: orotate phosphoribosyl transferase
OroP: orotate permease
PAGE: polyacrylamide gel electrophoresis
PDB ID: protein data bank identification
RNA: ribonucleic acid
SAX: strong anionic exchange
SEM: standard error of the mean
TBE: trizma base/borate/EDTA
Tm: melting temperature
UMPS: uridine monophosphate synthase
UV: ultraviolet
ε: molar extinction coefficient

## 1 Introduction

**SCHEME 1:**
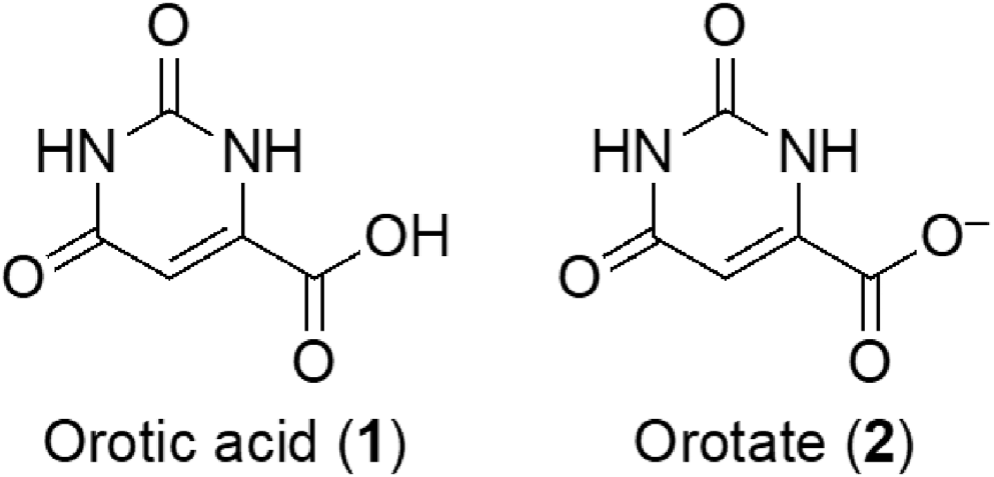
Structural representation of orotic acid (**1**) and orotate (**2**).

Orotic acid (OA, **1**, Scheme 1) and its anionic form, orotate (**2**), play a pivotal role in various biological processes, serving as an essential intermediate in pyrimidine *de novo* synthesis. This small molecule, with three distinct pK_a_ values at 2.8, 9.3, and 13, exhibits differential ionization corresponding to the carboxylic group and the two nitrogen atoms. In aqueous solution, OA demonstrates a dynamic behavior, transitioning between various forms based on pH conditions.

At pH 5–9, OA predominantly exists as the monoprotic anion orotate (**2**), while at pH > 9.3, it adopts the diprotic anionic form (Nath, Vats, and Roy 2013). The significance of orotate extends beyond its role as a precursor in pyrimidine nucleotide biosynthesis. It is actively taken up by erythrocytes and hepatocytes, contributing to uridine conversion and participating in the pyrimidine‐recycling pathway. Noteworthy associations include the link between dietary orotate and conditions such as fatty liver in rats and the potential mitigation of neonatal hyperbilirubinemia. Beyond these roles, orotate derivatives have shown promise as antipyrimidine drugs and complexes with metal ions and organic cations hold potential for therapies targeting metabolic syndrome (Löffler, Carrey, and Zameitat 2015). As a precursor of pyrimidine nucleotides, orotate finds applications in the food, pharmaceutical, and cosmetic industries. Recent efforts have been directed at enhancing its production efficiency, with engineered *Escherichia coli* demonstrating impressive results in OA synthesis (Li et al. 2022). The nutritional significance of OA is underscored by its presence in milk and dairy products, with historical recognition as “vitamin B13.” Its potential as a growth enhancer in livestock nutrition and its industrial production underscore its versatile applications. Experimental studies highlight OA's neuroprotective effects, improved learning behavior, and cardiovascular benefits. However, challenges in bioavailability have led to the development of pharmaceutical derivatives. OA's impact on lipid metabolism, hypocholesterolemic activity, and uricosuric effects have yielded mixed results, emphasizing the complexity of its interactions with gene expression (Löffler, Carrey, and Zameitat 2016). The historical roots of OA usage trace back to its discovery in whey in 1905. Initial findings highlighted its abundance in milk and dairy products, sparking interest in its potential nutritional significance. Subsequent investigations revealed variations in OA content in human and bovine milk, suggesting postsecretory changes. While early interest focused on OA's application in livestock nutrition, its pharmacological potential in humans gained traction. Experimental studies displaying OA's cardiovascular benefits fueled explorations into novel drug applications, while recent advancements in biomedical engineering have led to the development of innovative microvessel models crucial for cardiovascular research (Pitingolo et al. 2017), potentially offering platforms for studying orotate's impact on vascular health. OA's critical role in pyrimidine biosynthesis positions it as a key regulator of gene transcription, influencing cell and tissue development (Pulletikurti et al. 2022). Emerging research sheds light on OA's potential role in gene transcription regulation across different organisms. Animal models hint at the translation of embryonic phenotypes into human inborn errors of pyrimidine synthesis. As an alternative to uridine supplementation, OA or its precursor dihydroorotate (DHO) may hold promise in cases where the *de novo* pyrimidine‐biosynthetic pathway is defective or experimentally inhibited (Pacholko and Bekar 2023). Magnesium orotate has garnered attention in medical literature as a potential adjuvant in pediatric and adult gastroenterological disorders linked to dysbiosis. Studies explored its application in psychiatric disorders' treatment, particularly in major depression and anxiety. They revealed the efficiency of magnesium orotate therapy in cases presenting both gastroenterological and psychiatric symptoms, emphasizing the potential modulation of the gut–brain axis and its impact on patients with dual digestive and psychiatric symptoms (Schiopu et al. 2022). As for the enzymes using orotate as substrate, orotate phosphoribosyl transferase (OPRT), a bifunctional enzyme in mammalian cells, is a key player in pyrimidine biosynthesis. A novel fluorescence method utilizing 4‐trifluoromethylbenzamidoxime enables the measurement of OPRT activity in living cells, offering valuable insights into pyrimidine metabolism research (Shibata et al. 2023). Orotate is facilitated across cellular membranes by dedicated transporters, like orotate permease (OroP), pivotal for its utilization in pyrimidine nucleotide biosynthesis. These transporters are crucial in regulating cellular sensitivity to compounds like 5‐fluoroorotate, influencing nucleotide metabolism (Solem et al. 2008). Orotate, along with its derivatives such as 5‐fluoroorotate, not only serves as a precursor in pyrimidine nucleotide biosynthesis but can also function as an inhibitor of key enzymes in this pathway. Inhibition studies with orotate and its derivatives have demonstrated their potential as competitive inhibitors, particularly against enzymes like dihydroorotase and dihydroorotate dehydrogenase (DHODH), suggesting a promising avenue for the development of antimalarial drugs targeting the pyrimidine biosynthetic pathway, with both orotate and its derivatives showing efficacy in reducing parasitemia in *P. berghei*–infected mice (Krungkrai, Krungkrai, and Phakanont 1992). Several studies link human Miller syndrome, a rare genetic disorder primarily affecting facial, limb, and body development, to defects in the DHODH gene and, hence, to depleted orotate production. Another defect in pyrimidine biosynthesis, orotic aciduria, is found in humans and cattle with a deficiency of uridine monophosphate synthase (UMPS) (Nath, Vats, and Roy 2013). Thus, orotate therapy holds promise for addressing pyrimidine biosynthesis deficiencies in conditions such as those described above.

The intricate relationship between orotate and neurotherapy was also explored, including an examination of lithium orotate (LiOr) and its impact on neurological disorders such as bipolar disorder (BD). While lithium carbonate (Li_2_CO_3_) is effective in managing BD, it has associated adverse effects. LiOr emerged as a potential alternative due to its purported, enhanced blood–brain barrier permeability (Pacholko and Bekar 2021). In the realm of neurotherapeutics, an investigation was conducted into the role of orotate in addressing glucose hypometabolism, mitochondrial dysfunction, and cholinergic deficits characteristic of early‐stage Alzheimer disease (AD), utilizing the TgF344‐AD rat model. This model, which exhibited diminished activities in mitochondrial complexes, particularly in aged rats, was administered a nutrient cocktail comprising magnesium orotate, benfotiamine, folic acid, cyanocobalamin, and cholecalciferol. Remarkably, this cocktail effectively restored mitochondrial activities and augmented hippocampal acetylcholine levels under physiological stimulation. These findings underlined not only the potential utility of the TgF344‐AD rat model in elucidating mitochondrial and cholinergic dysfunction in AD but also the importance of exploring prospective treatment modalities centered around orotate supplementation (Viel et al. 2021).

One of the identified genetic factors underlying neurological disorders, such as amyotrophic lateral sclerosis (ALS) and frontotemporal dementia (FTD), involves a significant expansion of GGGGCC (G_4_C_2_) repeats within the *C9orf72* gene (Brčić and Plavec 2017). The pathological manifestation of these disorders is postulated to be intricately linked to the adoption of noncanonical structures, specifically G‐quadruplexes (G4), by the expanded repeats. While the typical normal range of G_4_C_2_ repeats is 2 to 24 copies, affected individuals harbor several hundreds or even thousands of repeats, constituting the primary genetic causation for familial ALS and FTD and a prevalent factor in sporadic ALS (Renton et al. 2011). The propensity of these expansion‐prone repeats to adopt stable secondary structures is considered pivotal in mechanisms governing repeat expansion or deletion. Recent investigations indicate that the presence of expanded G_4_C_2_ repeats perturbs DNA replication of the *C9orf72* gene, potentially leading to further expansion and disease progression (Van Blitterswijk, DeJesus‐Hernandez, and Rademakers 2012; Cruts et al. 2013). Besides DNA, RNA sequences containing G_4_C_2_ repeats have also been demonstrated to adopt noncanonical structures, including G4 (Brčić and Plavec 2018; Liu et al. 2021). While the precise mechanism through which expanded G_4_C_2_ repeats induce neurodegeneration remains elusive, three non‐mutually exclusive hypotheses have been proposed, involving loss of function of *C9orf72*, toxic RNA transcripts from the expanded DNA repeat, and the production of toxic dipeptide repeat proteins. The exploration of the G4 structuration of DNA oligonucleotides, specifically those comprising four G_4_C_2_ repeats, serves as a crucial avenue for unraveling the intricate mechanisms underlying neurological disorders, suggesting that molecules capable of modulating the process of G4 aggregation could hold significant potential for advancing neurotherapy approaches. This represents a novel perspective on biomolecular aggregation at the nanoscale, particularly pertinent to neurodegeneration, challenging the established paradigm positing that peptide (amyloid beta) and protein (tau) aggregation mainly govern the molecular events underlying neurological disease (Nisbet et al. 2015). In‐depth studies on oligonucleotides with G_4_C_2_ repeats have revealed a structural heterogeneity, with certain sequences forming multiple G4 structures. Notably, efforts to reduce this heterogeneity through nucleotide substitutions and sequence modifications have led to the identification of stable G_4_C_2_‐based G4 structures. The replacement of a single guanine with an 8‐bromoguanine in the sequence further demonstrated the potential to stabilize G4 structures, shedding light on the structural polymorphism intrinsic to G_4_C_2_ repeats. The study of such a DNA model contributed to a comprehensive understanding of the role of non‐canonical DNA structures in the pathogenesis of ALS and FTD, opening avenues for targeted therapeutic interventions (Brčić and Plavec 2017; Plavec 2020). Herein, we focus on d[(GGGGCC)_3_GGGG], a DNA model capable of forming G4 with G_4_C_2_ repeats that has been associated with neurodegeneration. Using various spectroscopic techniques (CD, UV, and thermal denaturations), PAGE electrophoresis, and molecular docking, our study examines the impact of orotate on the aggregation of this DNA associated with neurodegeneration as described in the sections below. Orotate, a naturally occurring pyrimidine derivative involved in nucleotide metabolism, is a small, planar molecule with the potential to interact with nucleic acid structures, including G4, through hydrogen bonding, stacking interactions, and electrostatic forces. Based on this, we speculated that orotate could modulate the properties of G4, potentially disrupting these structures in ways that could have therapeutic implications, as suggested by its emerging role in neurotherapy. This is the focus of the present work, where we aim to explore orotate's potential to influence G4 properties, particularly in the context of neurodegenerative diseases associated with G_4_C_2_ repeat expansions, as discussed in the following sections.

## 2 Experimental

### 2.1 DNA Synthesis and Purification

The d[(GGGGCC)_3_GGGG] oligodeoxynucleotide (ODN) was synthesized on a CPG resin under the standard β‐cyanoethyl phosphoramidite chemistry at a 15 μmol scale, using 3′‐phosphoramidite nucleosides on a Perseptive Biosystems (Framingham, MA, USA) Expedite DNA synthesizer. Subsequently, the solid support underwent a treatment with concentrated aqueous ammonia at 55°C for 7 h. The resulting solution was filtered, concentrated under reduced pressure, and subjected to purification by high‐performance liquid chromatography (HPLC) using a Nucleogel SAX 1000–8/46 anion exchange column (Macherey‐Nagel, Düren, Germany). A linear gradient of Buffer B in Buffer A was applied (ranging from 0% to 100% over 30 min). Buffer A consisted of 20 mM NaH_2_PO_4_ at pH 7.0, with 20% CH_3_CN, while Buffer B comprised 1 M NaCl, 20 mM NaH_2_PO_4_ at pH 7.0, and 20% CH_3_CN. The flow rate was maintained at 1 mL/min. After purification, the dried products were desalted using a BioGel P2 gel filtration column (Bio‐Rad Laboratories, Hercules, CA, USA) eluted with H_2_O and ethanol (9:1, v/v). The resulting pure sample was subsequently lyophilized. The quantification of the ODN was carried out spectrophotometrically in water at λ = 260 nm at 90°C, using a Jasco V‐530 spectrophotometer (JASCO Europe, Cremella, Italy). The quantification was accomplished utilizing a molar extinction coefficient (ε) of 204400 M^−1^ cm^−1^. ESI‐MS (m/z) calcd. for [M + K – 7H]^6−^ 1163.7 (expected) and 1162.0 (found); [M + K – 8H]^7−^ 997.3 (expected) and 995.9 (found); [M + K – 9H]^8−^ 872.5 (expected) and 871.4 (found); [M + K – 10H]^9−^ 775.4 (expected) and 774.4 (found); and [M + K – 11H]^10−^ 698.8 (expected) and 696.9 (found).

### 2.2 Sample Preparation

Prior to conducting structural and interaction analyses between DNA and orotate, the oligonucleotide sample was annealed to assume its secondary G4 conformation. A 10 mM KH_2_PO_4_ buffer containing 100 mM potassium chloride (KCl) at pH 7.0 was added to the lyophilized oligonucleotide to get a 1 mM stock solution. Subsequently, the ODN sample underwent heating to 90°C for 10 min, followed by rapid cooling to 4°C. Afterward, the sample was stored at 5°C for at least 24 h and allowed to equilibrate at 25°C for 2 h before each analysis.

### 2.3 CD and UV Experiments

**FIGURE 1:**
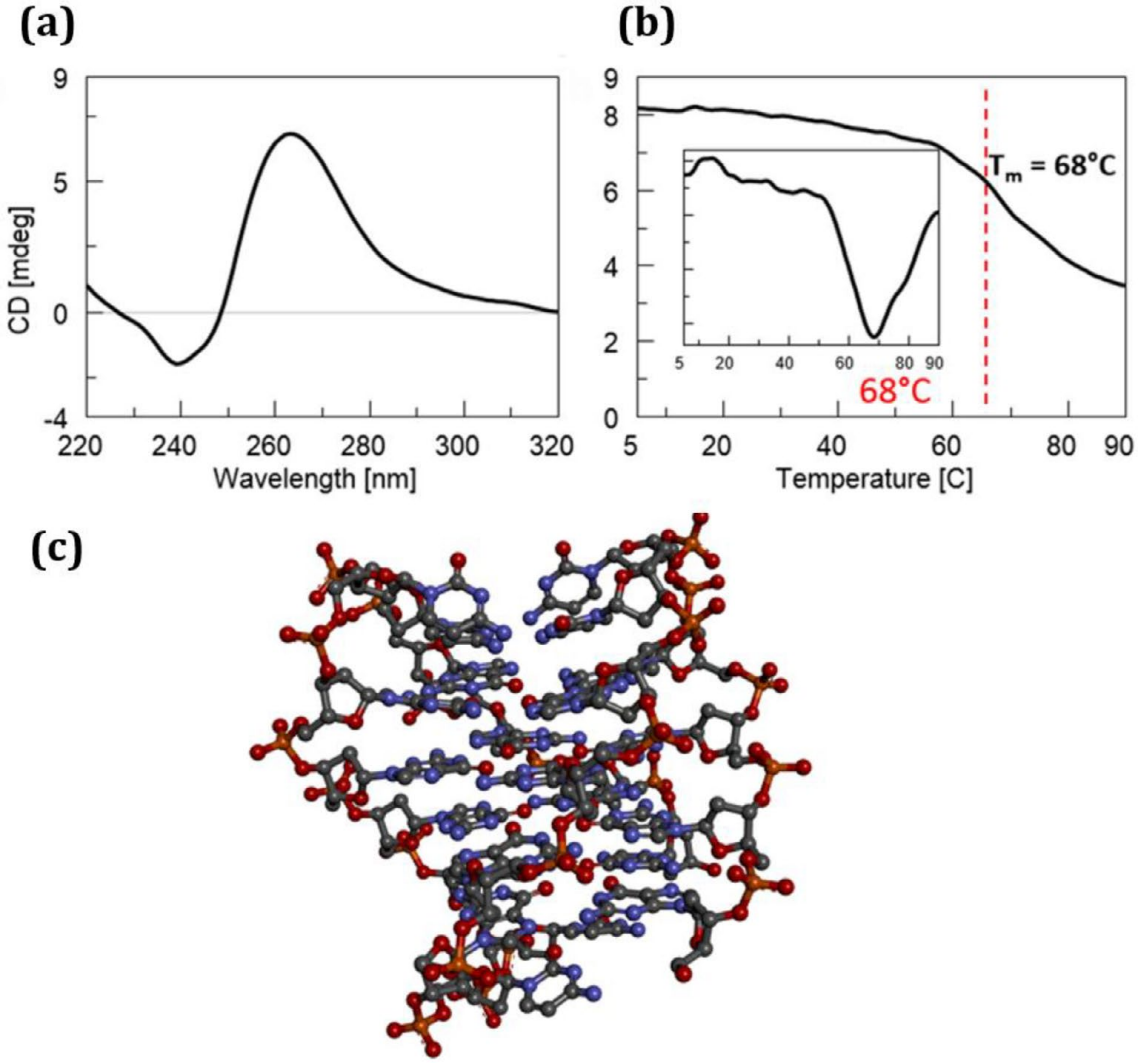
CD and CD melting analysis of d[(GGGGCC)_3_GGGG] DNA sequence annealed in 10 mM KH_2_PO_4_ buffer containing 100 mM KCl at pH 7.0, panels (a) and (b), respectively. The inset in (b) shows the first derivative of the CD melting curve used to determine the G4's T_m_. A three‐dimensional depiction of the antiparallel G4 topology assumed by the here studied DNA under thermodynamic annealing conditions is shown in (c).

**FIGURE 2:**
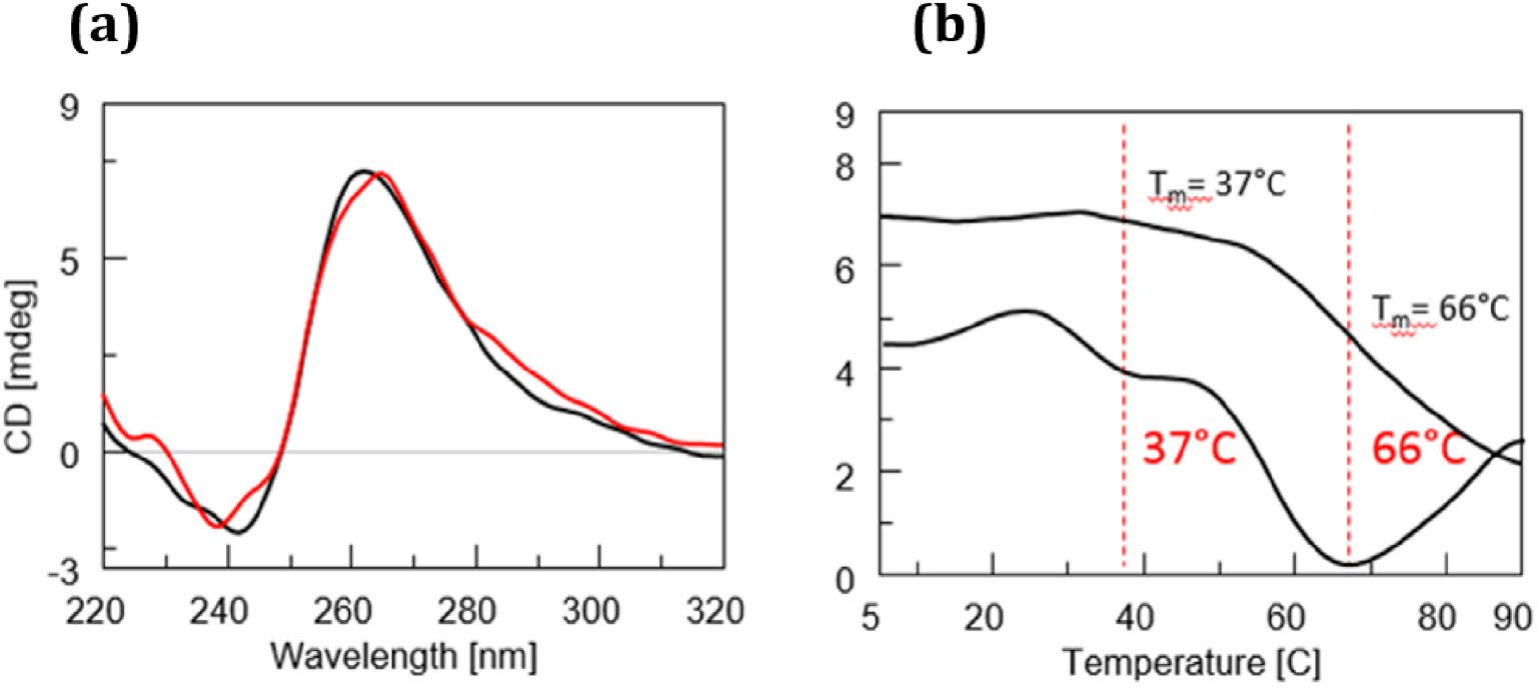
(a) CD analysis of d[(GGGGCC)_3_GGGG] DNA sequence annealed in 10 mM KH_2_PO_4_ buffer containing 100 mM KCl at 7.0 pH recorded before (black curve) and after (red curve) incubation with 10 equiv. OA. (b) CD melting and first derivative CD melting curves of G4 DNA complexed to the orotate ligand.

The CD spectra and CD melting profiles were acquired as previously reported (Zarrilli et al. 2017; Marzano et al. 2024) within the wavelength range of 220–320 nm at 25°C, utilizing quartz cuvettes with a 0.1 cm optical path in a 10 mM KH_2_PO_4_ buffer containing 100 mM potassium chloride (KCl) at pH 7.0, at the final single‐strand ODN concentration of 20 μM. All CD spectra were averaged over four scans, recorded at a scanning speed of 200 nm/min, with a response time of 1 s and a bandwidth of 2 nm. The CD spectra in Figures 1 and 2 were corrected by subtracting the buffer CD contribution. CD melting experiments were performed by monitoring the CD value of the higher positive Cotton effect within the temperature range of 5°C–90°C at a heating rate of 0.5°C/min. UV spectra were collected within the 220–320 nm range using quartz cuvettes with a 1 cm optical path in a 10 mM KH_2_PO_4_ buffer containing 100 mM potassium chloride (KCl) at pH 7.0. A 1 mM orotate solution was prepared from a 100 mM stock solution for the CD and UV experiments. The DNA sample was treated with orotate by directly adding the ligand solution into the solution containing the DNA. A waiting time of 5 min was observed after each addition before acquiring the UV and CD spectra. The CD experiments were performed after the complex formation, specifically when 10 equivalents of the ligand had been added. The CD melting analysis was performed at a rate of 0.5°C/min, in the temperature range 5°C–90°C. All CD and UV spectra were recorded using a JASCO 1500 spectropolarimeter equipped with a JASCO PTC‐348‐WI temperature controller.

### 2.4 PAGE Analysis

Native gel electrophoresis experiments were conducted using 18% polyacrylamide gels containing 1× TBE buffer (8.9 mM Trizma base, 8.9 mM borate, and 0.2 mM EDTA) and 30 mM KCl at pH 7.0, maintained at 5°C, and subjected to a voltage of 120 V for 2 h. Each sample, comprising 1 nmol, was diluted to 10 μL using a loading buffer containing 10% glycerol to aid sample loading into the wells. The gels were treated with SYBR Green (Merck KGaA, Darmstadt, Germany) and visualized using the Bio‐Rad Gel Doc TM XR apparatus (Bio‐Rad Laboratories, Hercules, CA, USA) at 260 nm.

### 2.5 In Silico Studies

The G4 model of the GGGGCC‐containing DNA was generated using the Discovery Studio (DS) 2021 software (Accelrys, San Diego, CA, USA), which was utilized for all visualizations in this study. The d[(GGGGCC)_3_GGGG] G4 was constructed based on the NMR structure with PDB ID: 2N2D (d[(GGGGCC)_3_GG(Br)GG]) (Brčić and Plavec 2015), from which the bromine atom was manually replaced with a hydrogen atom. Subsequently, energy minimization was performed using the CHARMm force field, following protocols described in previous studies (Ahmed, Anwar, and Thet Htar 2017).

The 3D structure of orotate was obtained from PubChem (https://pubchem.ncbi.nlm.nih.gov/) as a .sdf file, which was then converted to a .pdb file and analyzed using DS. The HDOCK program was employed for the *in silico* oligomerization of the G_4_C_2_‐containing DNA and for blind dockings with the ligand. This software utilizes the ITScore‐PP iterative knowledge‐based scoring function to rank the Top 10 poses resulting from the docking simulations. The program generates an energy score, referred to as the HDOCK score, which is dimensionless. Lower values of this score indicate stronger binding interactions between the molecules involved, which has been shown to correlate strongly with experimental binding affinities (Huang and Zou 2008). We focused on the highest‐ranked pose (Top 1) among the complexes predicted by HDOCK based on the energy scores provided by the program.

### 2.6 Cell Cultures and Treatments

Cell cultures were set up as previously described (Huang and Zou 2008). Human SH‐SY5Y cells were grown as monolayers in a Dulbecco's modified Eagle's medium (DMEM) supplemented with 10% FBS, 1% penicillin (50 IU/mL), and streptomycin (50 μg/mL) in a humidified atmosphere at 37°C with 5% CO_2_. The cells were plated on multiwells at the density of 50000 cells/mL and exposed to 10 μM retinoic acid to induce a neuronal phenotype. Once differentiated, the cells were treated with OA for 24 h (10 mM in DMSO) to the final concentrations of 0.1, 1.0, and 10 μM. The cells were also treated with only DMSO at the highest concentration (1 μL/mL) as a negative control.

### 2.7 MTT Assay

Mitochondrial activity was assessed by the MTT (3[4,5‐dimethylthiazol‐2‐yl] 2,5‐diphenyl‐tetrazolium bromide) assay, as previously reported (Piccialli et al. 2020). Briefly, after treatments, neuronal cells were incubated with an MTT solution (1 h at 37°C). In viable cells, the mitochondrial dehydrogenases convert the MTT reagent into water‐insoluble formazan crystals. At the end of the incubation, insoluble crystals were dissolved in DMSO, and the absorbance was determined spectrophotometrically at 540 nm. The data are expressed as a percentage of mitochondrial activity relative to control values.

### 2.8 Statistics

Statistical analyses were conducted using GraphPad Prism 8.0 (GraphPad Software, La Jolla, CA). Data are presented as mean ± SEM from individual experiments. To compare groups, one‐way analysis of variance (ANOVA) was performed, followed by the Bonferroni post hoc test to adjust for Type I error in multiple comparisons; a *p*‐value of < 0.05 was deemed statistically significant. No outlier tests were applied, and normality of the data was not assessed.

## 3 Results and Discussion

### 3.1 Experimental Studies on the Impact of Orotate on d[(GGGGCC) _3_GGGG] G4 Structure and Stability

**FIGURE 3:**
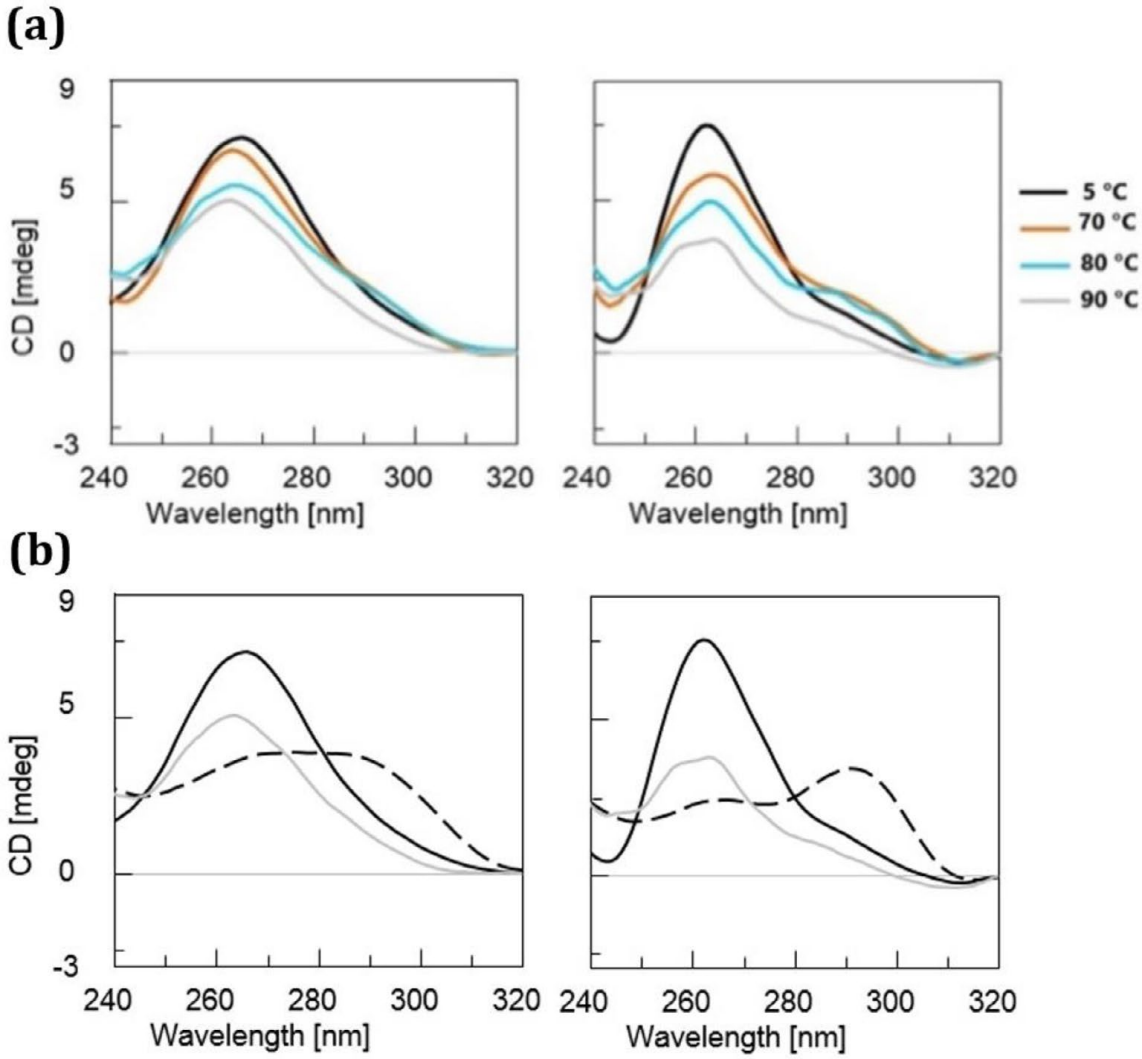
CD analysis of d[(GGGGCC)_3_GGGG] DNA sequence annealed in 10 mM KH_2_PO_4_ buffer containing 100 mM KCl at pH 7.0 alone (left) and in complex with orotate (right). (a) CD curves recorded at 5°C and after heating the samples at the specified temperatures. (b) CD curves recorded at 5°C before heating (black line), 90°C (gray line), and 5°C after heating (dashed line).

G4s represent non‐canonical secondary structures of nucleic acids that can arise within guanine‐rich regions of regulatory genomic sequences in both human and viral genomes. Within this framework, ligands targeting G4 structures have been synthesized and evaluated for their therapeutic potential (Scuotto et al. 2014; Falanga et al. 2019; Platella et al. 2021, 2020; Pirota et al. 2021; Riccardi et al. 2020). In this study, we delved into the experimental investigation of a DNA model derived from the *C9orf72* gene, recognized for housing the hexanucleotide sequence GGGGCC, which, as mentioned above, is associated with neurodegenerative diseases. The main aim of this study was to assess orotate's ability to modulate the G4 structures formed by the d[(GGGGCC)_3_GGGG] sequence, which was synthesized *via* solid‐phase synthesis utilizing 3′‐phosphoramidite chemistry. The identity of the synthesized sequence was validated using electrospray ionization mass spectrometry (ESI MS, Supporting Information S1, Figure S1, Table S1). A range of analytical methods were employed to explore the characteristics of this GGGGCC‐containing G4 DNA model and its interaction with orotate. These techniques included circular dichroism (CD) spectroscopy, which is used for both CD binding assays and melting analysis, UV spectroscopy, and electrophoretic mobility assays. In particular, CD and UV are commonly employed techniques for examining the binding affinity of oligonucleotides and their analogs with biomedically significant targets (Zarrilli et al. 2017). Through these methods, we aimed to gain insights into the DNA sequence's structural properties and the impact of orotate on its stability and conformation (Figures 1 and 2). Figure 1 illustrates the CD and CD melting analyses of the d[(GGGGCC)_3_GGGG] sequence annealed under kinetic control in KH_2_PO_4_ buffer containing 100 mM potassium chloride at pH 7.0. The CD analysis shows the presence in solution of a parallel DNA G4 structure, as evidenced by the negative band around 240 nm and the positive band centered around 265 nm (Kan et al. 2007; Grande et al. 2017). Thus, remarkably, the DNA initially assumed a parallel G4 conformation upon sample preparation and CD analysis. However, following the melting process, which unveiled a melting temperature (T_m_) of 68°C (Figure 1b) and subsequent slow cooling, the quadruplex transitioned to an antiparallel structure (Figure 1c), as indicated by the emergence in the CD spectrum of a distinct shoulder centered around 300 nm (Figure 3). These experiments confirmed the dynamic nature of this neurological disease‐associated DNA, showcasing its capability to adopt diverse topologies depending on both experimental and physiological conditions. Subsequently, the CD analysis was replicated following the addition of orotate as a ligand, employing a similar methodology (Figure 2). To rule out aggregation phenomena within the orotate solutions used for the binding study, we recorded the UV spectra of increasing concentrations of pure orotate dissolved in the G4 buffer. Remarkably, the resulting absorbance (at 280 nm) *vs* concentration trend (Figure S2a) demonstrated linearity, thus indicating that orotate did not aggregate within the concentration range utilized in the binding assays presented in this study. On the other hand, the UV absorbance at 260 nm *vs* concentration plot, between 0 and 3 μM, shows an interaction between the ligand and G4 DNA (Figure S2b). The sigmoidal shape suggests cooperative binding, with the half‐maximum absorbance concentration, related to the binding constant, at approximately 1.3 μM, indicating effective binding. At 4–6 μM, the absorbance increases linearly with concentration (Figure S2c), which may imply nonspecific interactions or saturation effects. However, from 7 to 10 μM, a second sigmoidal increase in absorbance suggests a potential secondary binding phase or conformational change in the G4 DNA upon higher ligand concentrations (Figure S2c). Kinetic experiments show that the complex formed by orotate and d[(GGGGCC)₃GGGG] DNA does not change significantly in its UV spectra over time (Figure S3a)

CD melting analysis performed on the DNA/orotate incubates revealed the presence of multiple melting points within the denaturation process, notably evident at the temperatures of 37°C and 66°C, unveiling the impact of orotate on the thermal stability of the G4‐folded DNA (Figure 2). As for the specificity of orotate binding, we expanded our study to include the human Tel22 (d[AGGGTTAGGGTTAGGGTTAGGG]) sequence (Terenzi et al. 2014), which forms in telomeric regions. The first derivative analysis of CD melting curves, presented in the Supporting Information S1 (Figure S3b), revealed no shift in the melting temperature of Tel22 upon orotate addition, suggesting that orotate's stabilizing effect may be selective for specific G4 sequences, such as GGGGCC, rather than a general feature across all G4. Besides its effect on the G4 thermal stability, the addition of orotate did not induce significant structural changes in the G4 folding, as evidenced by the nearly unchanged CD profile following complexation with the ligand (red line, Figure 2a). However, orotate binding exerted significant effects on the quadruplex's propensity to undergo denaturation, as evidenced by the CD analysis shown in Figure 3. While the CD curves of G4 in the absence of orotate showed relatively limited variation in the temperature range of 5°C–90°C (Figure 3a, left), the addition of the orotate ligand led to more pronounced changes in the CD profiles recorded at increasing temperatures (Figure 3a, right), indicating alterations in the DNA secondary structure caused by the ligand. Notably, the presence of the orotate ligand during the cooling process yielded a different CD spectrum profile (Figure 3b, dashed lines), demonstrating the ability of orotate to induce conformational changes in the studied neuropathology‐associated DNA sequence. This interaction appeared to influence the DNA's ability to form alternative structures. After the complex was formed, there were no significant kinetic modifications observed, as indicated by the kinetic experiments depicted in Figure S3.

The lack of overlap between the CD curves at 5°C before and after the thermal treatment, as observed in Figure 3b, indicates that the heating–cooling process is not reversible for the G4 DNA investigated in this study. This irreversibility suggests that the conformational changes induced by the thermal treatment are not restored upon cooling, both in the unliganded DNA and in the complex with orotate. This highlights the dynamic and intricate nature of the G_4_C_2_‐rich G4 structures. Moreover, the presence of orotate appears to enhance the prevalence of the antiparallel quadruplex formation, as indicated by the CD spectrum illustrated in Figure 3b (dashed line, right panel), featuring a pronounced peak at approximately 290 nm. This contrasts with the mixture of topologies observed in the absence of orotate during the cooling process, as depicted by the CD profile obtained after cooling (Figure 3b, dashed line, left panel), where a shoulder around 280–290 nm is evident, rather than a distinctly separate band as observed in the presence of orotate.

**FIGURE 4:**
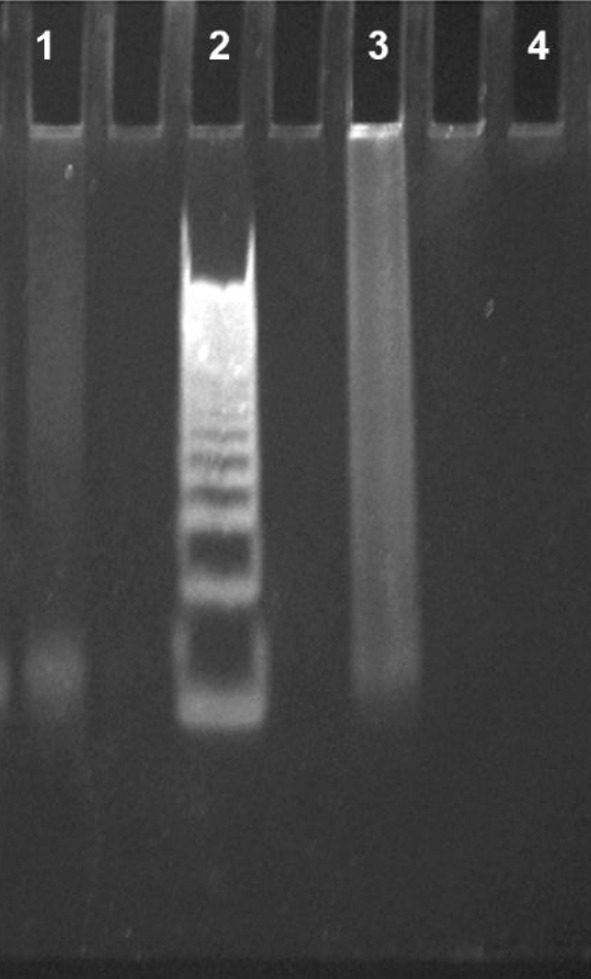
Non‐denaturing polyacrylamide gel electrophoresis (PAGE) in TBE 1x at pH 7.0 of d[(GGGGCC)_3_GGGG] G4 alone (Lane 3), G4 incubated with 10 equivalents of orotate (Lane 1), and orotate (Lane 4). The 10–100 bp DNA ladder was used as the reference control (Lane 2). The gel was stained with SybrGreen and visualized by UV irradiation at 260 nm.

Similarly, the PAGE experiment (Figure 4, Figure S4) revealed for the untreated DNA sequence with orotate an extended, broadband rather than a clear band, suggesting, as also observed *via* CD, that this DNA involved in neuropathologies is capable of forming a mixture of structures (Lane 3). Following treatment with orotate, a single prevalent band with a molecular weight consistent with monomeric 22‐mer was detectable (Lane 1), which is in line with the results shown in Figure 3b, indicating the selection of a primary structure after orotate treatment. Overall, the CD analysis confirmed the formation of G4 structures by the d[(GGGGCC)_3_GGGG] G‐rich DNA under our experimental conditions. Initially assuming a parallel conformation, the G4 transitioned also to an antiparallel structure upon melting and subsequent cooling, showcasing the dynamic nature of the neurological disease‐associated DNA used in this study. Upon addition of orotate, CD melting analysis revealed multiple melting points within the denaturation process, signifying orotate's impact on DNA stability, notably evident at temperatures of 37°C and 66°C.

Considering that a unique T_m_ of 68°C could be detected in the absence of orotate, the observed destabilizing effect was due to the interaction with the ligand. While the initial denaturation process did induce significant but less intense structural changes in the DNA, orotate binding significantly affected the quadruplex's propensity to undergo denaturation, as evidenced by more pronounced changes in CD profiles at higher temperatures. Moreover, the different CD spectra obtained upon cooling the DNA in the presence of orotate revealed the ability of orotate to influence the DNA's ability to form alternative structures after thermal treatment. Further support for this hypothesis came from PAGE evidence. The incubation with orotate resulted in a single prevalent band (Figure 4, Lane 1) whose migration was consistent with a monomeric 22‐mer DNA sequence, highlighting the selection of a primary structure following orotate treatment, which agrees with the observed trend in Figure 3b. These findings underscore the dynamic interplay between orotate and the secondary structures assumed by the d[(GGGGCC)_3_GGGG] DNA sequence, suggesting the potential therapeutic implications of this food supplement in neuropathy‐related conditions.

### 3.2 Computational Studies on the d[(GGGGCC) _3_GGGG] G4 and its Complexes With Orotate

The investigation into the interaction between the G4 formed by the d[(GGGGCC)_3_GGGG] DNA and orotate involved a multifaceted approach combining computational modeling and the above‐described experimental studies. As for the computational investigation, the initial step included building a model for the neuropathology‐related G4 based on the NMR structure (PDB ID: 2N2D). This structure corresponds to the DNA d[(GGGGCC)_3_GG(Br)GG], in which the bromine atom was manually replaced by us with a hydrogen atom. Following this, energy minimization was carried out using the CHARMm force field. This model exhibited an antiparallel G4 structure, a prevalent conformation revealed in our experiments during the cooling phase following heating conditions and confirmed as the primary structure after binding with orotate, as indicated by CD studies (Figure 3). The G4 model served as the foundation for *in silico* experiments, notably docking studies with orotate and G4 DNA in both monomeric and tetrameric forms. Molecular docking, particularly self‐docking simulations, is a useful method for investigating the self‐assembly of molecular structures. In self‐docking, multiple copies of the molecule of interest are used, each acting as both a ligand and a receptor. This approach aims to identify the most energetically favorable molecular arrangements and to explore the forces driving their self‐assembly. A relevant example is the study by Scognamiglio et al. (2023), which examined the self‐assembly of a nucleobase‐bearing amino acid derivative through self‐docking simulations. This research highlighted the use of self‐docking to predict the molecular interactions involved in the self‐organization of nucleobase‐functionalized structures. Interestingly, molecular docking simulations were also used to study the self‐assembly of the amidated FEYNF peptide (Jitaru et al. 2024), as well as to model the self‐aggregation of WWgc fibers (Mosseri et al. 2023). In our study, the HDOCK software was utilized to conduct *in silico* oligomerization of the G_4_C_2_‐containing DNA and blind dockings with the ligand. This tool employs the ITScore‐PP iterative knowledge‐based scoring function to rank the Top 10 poses obtained from the docking simulations. It generates a dimensionless energy score known as the HDOCK score (Sargsyan et al. 2025). Decreased values of this score signify more robust binding interactions between the molecules under consideration, a trend consistently associated with experimental binding affinities (Huang and Zou 2008). Monomeric antiparallel G4–ligand docking revealed a favorable binding process, supported by a negative score of −82.22, indicating a stable complex formation (Table 1, Figure S5). While the affinity for the parallel form of G4 DNA is predicted to be slightly higher (−95.24 vs. –82.22, Figure S5), as indicated by HDOCK docking with the Pu22 (d[TGAGGGTGGGTAGGGTGGGTAA]) sequence (Ambrus et al. 2005), a shorter, mutated analog of the G4‐forming Pu27 sequence in the c‐myc promoter (Dang 2012), herein used as a model of parallel G4 (PDB ID: 1XAV), the experimentally predominant form for the sequence studied in this paper is the antiparallel conformation, which was therefore used in the subsequent docking studies. Subsequent *in silico* oligomerization steps led to the creation of a tetramer G4 ((G4)_4_, Table 1). Symmetrical dimerization ((G4 + G4), Table 1) involving the open sides of two adjacent G4 structures resulted in a highly stable complex formation, as indicated by a score of −212.17 (Table 1). Further oligomerization steps involving bends/open interactions (Figure S5) produced energetically favored complexes, with scores of −117.79 and −115.06 (Table 1). These computational results suggested that the aggregation of the G4 into tetramers involves stable interactions. This docking study was consistent with experimental findings, suggesting a strong tendency for the G_4_C_2_‐containing DNA to oligomerization (Brčić and Plavec 2015). Interestingly, orotate binding to the G4 structures (Table 1) is predicted to diminish the association of additional G4 units, particularly evident in the case of dimer and tetramer formation as indicated in Table 1.

**TABLE 1:** HDOCK scores (Top 1 and means of the Top 1–3 scores ±S.D.) predicted for the formation of the G4 aggregates (dimer, trimer, tetramer) and of the complexes of orotate (L) with G4 monomer and tetramer.

Entry	HDOCK score Top 1	HDOCK score mean Top 1–3
G4 + G4	−212.17	−183.5 ± 30.2
**G4 + G4L**	**−208.26**	**−169.8 ± 34.3**
(G4)_2_ + G4	−117.79	−108.2 ± 10.1
(G4)_2_ + G4L	−118.31	−116.6 ± 1.5
(G4)_3_ + G4	−115.06	−113.60 ± 1.4
(G4)_3_ + G4L	−122.20	−118.0 ± 5.3
(G4G4L) + G4	−117.37	−112.8 ± 38.6
**(G4G4LG4) + G4**	**−74.14**	**−71.6 ± 3.5**
G4 + L	−82.22	−79.4 ± 2.6
(G4)_4_ + L	−109.29	−91.8 ± 15.8

*Note:* The red color refers to scores for dimer and tetramer formation in the absence of orotate, while the bold black color refers to scores in the presence of orotate.

**FIGURE 5:**
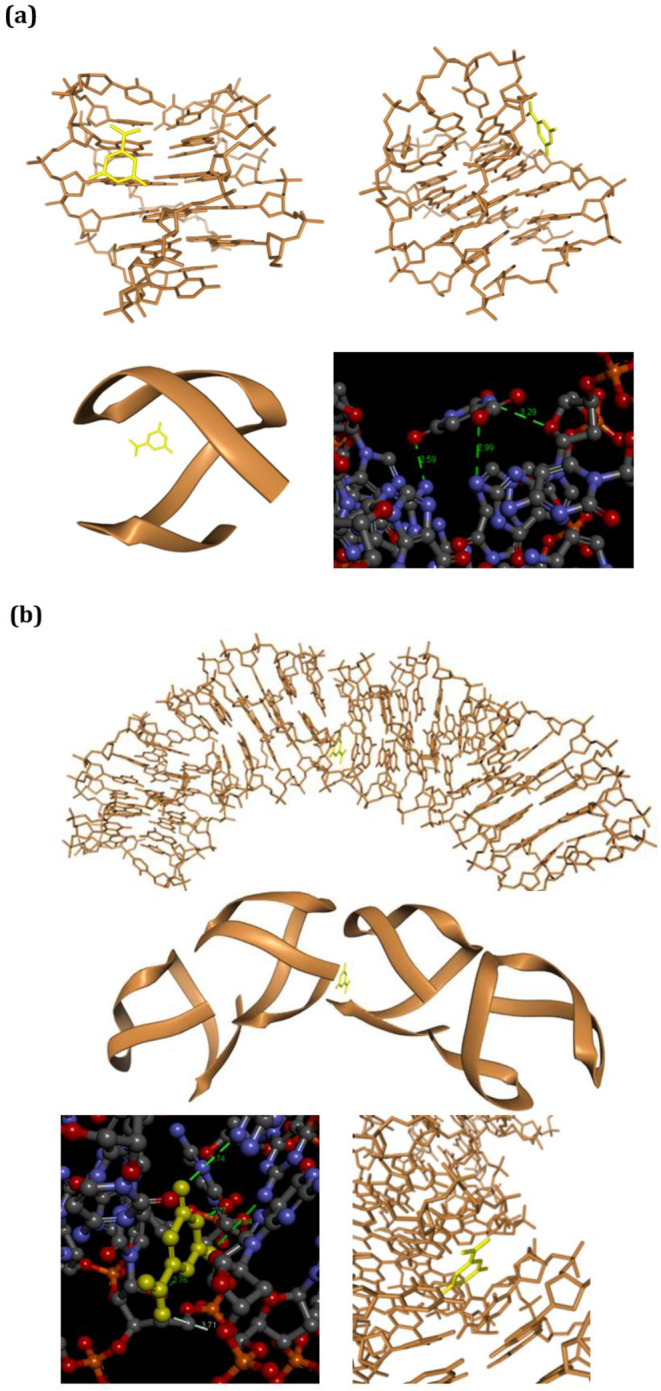
This figure illustrates molecular docking studies using orotate as a ligand and a model of the d[(GGGGCC)_3_GGGG] G4 in its monomeric (a) and tetrameric (b) forms. The ligand, highlighted in yellow for clarity, is depicted alongside various pose views, each offering different levels of zoom and showcasing the binding interactions (highlighted by green dashed lines for hydrogen bonds and white dashed lines for hydrophobic interactions) between orotate and the G4 systems. Notably, in (b) (bottom, right), the coplanarity of the orotate ring and the adjacent cytosine ring is evident, suggesting potential stacking interactions between the orotate and the nucleobase in the G4 tetramer.

A comparison of HDOCK scores for dimer and especially tetramer formation in the absence (red) and presence (bold black) of orotate reveals significantly lower scores for the latter complexes, both for the Top 1 pose and the averages of the first three poses (dimer: −208.26 *vs* –212.17; −169.8 ± 34.3 *vs* –183.5 ± 30.2; tetramer: −74.14 *vs* –115.06; −71.6 ± 3.5 *vs* –113.60 ± 1.4). In essence, computational studies suggest orotate as an inhibitor of G4 aggregation, a desirable characteristic in neurotherapeutic approaches for ALS and FTD. The ligand demonstrated a propensity to bind within the major groove of the G_4_C_2_‐containing DNA, forming three intermolecular hydrogen bonds (Figure 5a). In the orotate/tetramer docking, the ligand interacted with two G4 units inside the tetramer, forming a complex with a negative score of −109.29 (Table 1). This score indicated a higher tendency of orotate to bind the aggregated G4 than its monomeric form. The ligand's interaction involved three hydrogen bonds with one G4 unit and two additional interactions with the adjacent monomer (Figure 5b). The molecular docking suggested a stacking interaction for the orotate/aggregate interaction (Figure 5b), contrasting with the G4 monomer/orotate interaction inside the major groove (Figure 5a). The computational studies collectively revealed that orotate has a higher affinity for the aggregate form of the studied G4, resulting in structurally modified aggregates with disrupted intermolecular interactions.

Notably, the PAINS (Pan Assay Interference Compounds) score for both orotic acid and orotate was predicted to be 0, indicating no off‐targetalerts and suggesting a low likelihood of non‐specific interactions in biological assays (Figure S6). The transition from a parallel to an antiparallel G4 structure for the d[(GGGGCC)_3_GGGG] DNA in the presence of orotate upon melting and subsequent cooling is influenced by several factors, including temperature and the inherent flexibility of G4. At higher temperatures, the unliganded DNA begins to denature, and the G4 structure destabilizes. This leads to a mixture of structures and also to a partial transition from the parallel to an antiparallel form. In fact, upon cooling, the structure refolds into multiple topologies, and the antiparallel form is also detected, as indicated by a shoulder including a band in the CD spectra at around 290 nm. This change in conformation is likely due to the more unfavorable reformation of the parallel structure upon cooling, allowing for the stabilization of antiparallel topologies. The presence of orotate plays a crucial role in altering this process. Orotate binding to the G4 shifts the melting behavior, as evidenced by multiple melting points in the CD melting analysis, particularly at temperatures around 37°C and 66°C. This indicates that orotate destabilizes the G4 structure and modulates its thermal stability. Interestingly, orotate does not induce large‐scale changes in the overall folding of the G4 but rather affects its propensity to undergo denaturation. The CD profiles reveal that orotate stabilizes the antiparallel G4, as evidenced by the emergence of a distinct peak at 290 nm during the cooling phase, suggesting that orotate may help stabilize this conformation while destabilizing others. The transition from parallel to antiparallel G4 forms is thus influenced by both temperature and the binding of orotate, which modulates the structural equilibrium of the G4. The irreversibility of the heating–cooling process, observed in our experiments, suggests that the G4 undergoes permanent structural changes upon heating. This irreversibility could reflect the biological stability of G4 in cellular environments, where these structures may not easily revert to their original form after destabilization. This could have significant functional consequences, especially in the context of gene regulation, where G4 plays a role in controlling transcription and translation processes. The herein‐described computational studies provide further insight into the molecular basis of orotate's effects on the G4. The docking studies suggest that orotate reduces the aggregation of G4 by binding to the G4 units. This binding likely occurs through hydrogen bonds and van der Waals interactions, which impairs the formation of higher‐order aggregates, such as dimers and tetramers. Orotate thus stabilizes individual G4 units and interferes with the aggregation process, as confirmed by the decreased likelihood of aggregation predicted by us. When compared to other ligands known to interact with G4, many G4 ligands stabilize specific conformations or induce folding, but orotate's ability to influence both the thermal stability and the aggregation properties of neurodisease‐related G4 is an interesting feature. Moreover, orotate's interaction with the GGGGCC‐repeated DNA, which is associated with neurodegenerative diseases like ALS, positions it as a promising therapeutic candidate for targeting G4 in these diseases. Its capacity to destabilize GGGGCC‐containing G4, combined with its inhibition of their aggregation, makes orotate a unique and powerful ligand, especially in the context of diseases where such G4 structures play a critical role in pathogenesis.

### 3.3 Assessment of Mitochondrial Dehydrogenase Activity in Neuronal Cells Exposed to Orotate

As mentioned above, orotate might have therapeutic relevance in neurological disorders such as ALS, and FTD, characterized by the expansion of G_4_C_2_ repeats within the *C9orf72* gene. In a previous study, a cocktail of magnesium orotate nutrients was reported to restore mitochondrial respiration in the brains of TgF344‐AD and aged rats (Viel et al. 2021). Similarly, another mix of nutrients including magnesium orotate displayed effectiveness in counteracting mitochondrial dysfunction in AD neuronal cells (Babylon, Meißner, and Eckert 2023).

**FIGURE 6:**
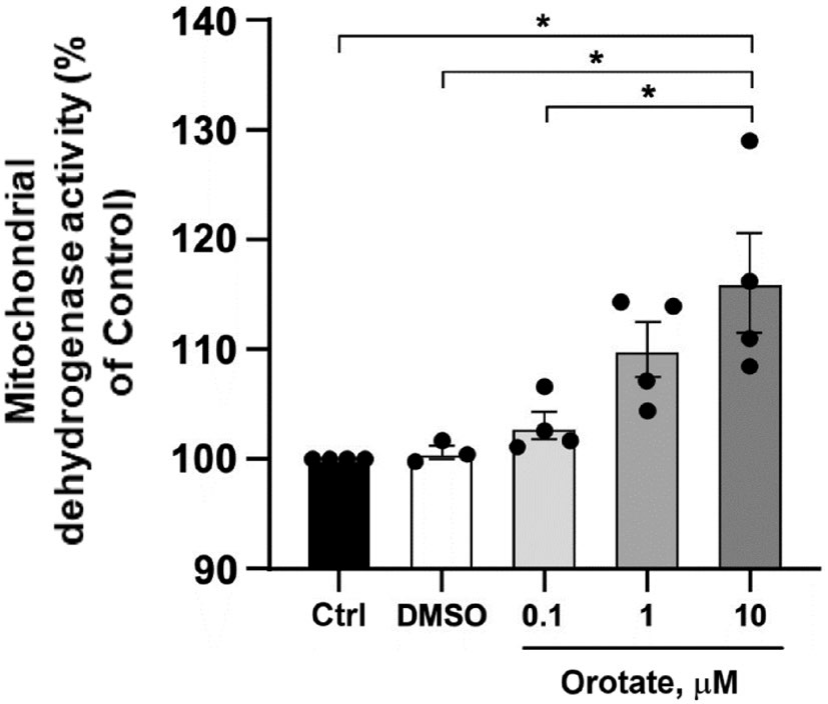
Evaluation of mitochondrial dehydrogenase activity in neuronally differentiated SH‐SY5Y cells treated with only DMSO and with 0.1, 1.0, and 10 μM orotate. Data are represented as percentage of control and expressed as mean ± S.E.M. of three independent experimental sessions, *p < 0.05 (df: 18, *F* value: 7.533, *p* value: 0.0019). The black dots indicate individual sample values (*n* = 3–4).

However, there is still no evidence about the effect of orotate alone on mitochondrial activity in neuronal cells in control conditions. For this reason, we have performed the MTT assay to assess any modulation of the mitochondrial dehydrogenase activity in neuronal cells exposed to orotate. To this aim, we treated neuronally differentiated human neuroblastoma (SH‐SY5Y) cells with orotate at the final concentrations of 0.1, 1.0, and 10 μM for 24 h and assessed the MTT reduction as an index of mitochondrial dehydrogenase activity at the end of the treatment (Figure 6).

Importantly, we did not find any cytotoxicity to be exerted by orotate, as no significant reduction of mitochondrial activity was observed in the orotate‐treated cells at any of the tested concentrations. On the other hand, we observed a slight, albeit statistically significant, increase in mitochondrial dehydrogenase activity in the cells treated with 10 μM orotate compared to control cells and to cells treated with only DMSO. In contrast, while a trend towards increased activity was observed at 0.1 and 1 μM orotate, statistical analysis did not suggest a significant modulation at these concentrations. Of note, this finding does not stray considerably from the previous results by Viel et al. (2021), which show that orotate alone could not significantly affect mitochondrial respiration. Nonetheless, orotate might exert its reportedly protective effect on cell metabolism downstream mitochondrial dehydrogenase activity as an intermediate of pyrimidine biosynthesis or, as suggested in the present study, by modulating DNA stability. Moreover, it cannot be ruled out that a more remarkable effect of orotate on cell metabolism might be observable in a pathological cellular context, in the presence of pronounced mitochondrial dysfunction and cell damage, or in combination with other nutrients, as reported previously (Viel et al. 2021).

## 4 Conclusions

The herein‐presented exploration of the G_4_C_2_‐based G4 aggregation, employing both *in silico* and experimental methodologies with a small molecule like orotate, an ingredient of food supplements commonly used and considered safe, offers valuable insights into potential therapeutic approaches for ALS and FTD. The convergence between computational and experimental evidence in our study underscores the orotate's binding to G4 structures, providing critical insights into the molecular intricacies of these interactions. More in detail, in this study, we investigated the impact of orotate on the G4 structures formed by the d[(GGGGCC)_3_GGGG] sequence as a model derived from the *C9orf72* gene. Our experimental approach encompassed various analytical methods, including CD spectroscopy, with both CD binding and melting analysis, UV spectroscopy, and electrophoretic mobility assays to elucidate the structural properties of the DNA sequence and the influence of orotate on its stability and conformation. Our findings confirmed the dynamic nature of this neuropathology‐associated DNA, with the d[(GGGGCC)_3_GGGG] initially assuming a parallel G4 conformation, transitioning to an antiparallel one upon melting and subsequent cooling. Orotate binding significantly influenced the quadruplex's propensity to undergo denaturation, increasing it, as evidenced by pronounced changes in CD intensity and alterations in spectroscopic profiles. Additionally, PAGE provided further evidence of orotate's impact on the G4 conformation. In the presence of orotate, a primary structure consistent with a monomeric 22‐mer was selected, contrasting with the mixture of G4 aggregates suggested by PAGE in the absence of orotate. Furthermore, our computational studies complemented the experimental findings by elucidating the interaction between the G_4_C_2_‐based G4 DNA and orotate at a molecular level. The docking simulations suggested that orotate reduces the aggregation of G4 units, particularly evident in dimer and tetramer formation. Interestingly, orotate demonstrated a higher affinity for the aggregate form of G_4_C_2_‐based G4 DNA, resulting in structurally modified aggregates with disrupted intermolecular interactions. In conclusion, orotate emerges as a potential candidate for neurotherapeutic approaches, exhibiting a destabilizing effect on neurological disease‐related G4 aggregates, thus highlighting its potential therapeutic significance in ALS and FTD. Additionally, the slight increase in mitochondrial dehydrogenase activity, as assessed by the MTT assay, underscores orotate's potential to enhance mitochondrial function, which is beneficial for its development as a neurodrug. While this study provides valuable insights into the binding and modulatory effects of orotate on neurodisease‐related G4 DNA structures, there are several limitations that warrant further exploration. First, the study was primarily conducted using in vitro models, which may not fully capture the complexity of biological systems. Future research should aim to explore orotate's effects in more complex biological models, such as cellular assays using different cell types, and in vivo systems to better understand its therapeutic potential and bioavailability. Additionally, the potential impact of orotate on genomic stability or other cellular pathways remains to be investigated. Expanding the study to include more diverse G4 sequences and examining its effects on gene expression and cellular function in relevant disease models, such as ALS or other neurodegenerative disorders, would provide a more comprehensive understanding of orotate's therapeutic potential.

## Author Contributions

**Andrea Patrizia Falanga:** investigation, validation, formal analysis. **Ilaria Piccialli:** investigation, validation, formal analysis. **Francesca Greco:** investigation. **Stefano D'Errico:** investigation, validation, formal analysis, visualization, writing – original draft, methodology. **Maria Grazia Nolli:** validation, investigation. **Nicola Borbone:** investigation, visualization, writing – original draft, writing – review and editing, validation, funding acquisition. **Giorgia Oliviero:** investigation, visualization, validation, methodology, funding acquisition. **Giovanni N. Roviello:** investigation, supervision, visualization, writing – review and editing, writing – original draft, conceptualization, methodology, data curation, formal analysis, validation.

## Conflicts of Interest

The authors declare no conflicts of interest.

### Peer Review

The peer review history for this article is available at https://www.webofscience.com/api/gateway/wos/peer‐review/10.1111/jnc.16296.

## Supporting information


Data S1.

## Data Availability

The data supporting the findings of this study are available within the manuscript and its Supporting Information S1 files. No additional datasets were generated or analyzed during the current study.
