# Supplementary material for: Nanostructural Modulation of G‐Quadruplex DNA in Neurodegeneration: Orotate Interaction Revealed Through Experimental and Computational Approaches
Source: J Neurochem. 2025 Jan 20;169(1):e16296. doi: 10.1111/jnc.16296 (PMC11744338; doi:10.1111/jnc.16296)
Supplement: Supplementary file 1 — Data S1. [file JNC-169-0-s001.pdf]

Electronic Supplementary Material

Nanostructural modulation of G-quadruplex DNA in neurodegeneration: orotate interaction revealed through experimental and computational approaches

Andrea Patrizia Falanga[1,¶], Ilaria Piccialli[2,¶], Francesca Greco[1], Stefano D'Errico[1] (✉), Maria Grazia Nolli[1], Nicola Borbone[1,3], Giorgia Oliviero[3,4], and Giovanni N. Roviello[5] (✉)

[1] *Department of Pharmacy, University of Naples Federico II, Via Domenico Montesano 49, 80131 Naples, Italy*
[2] *Division of Pharmacology, Department of Neuroscience, Reproductive and Dentistry Sciences, School of Medicine, University of Naples Federico II, Naples, Italy*
[3] *ISBE-IT, University of Naples Federico II, Corso Umberto I, 80138 Naples, Italy*
[4] *Department of Molecular Medicine and Medical Biotechnologies, University of Naples Federico II, via Sergio Pansini 5, 80131 Naples, Italy*
[5] *Institute of Biostructures and Bioimaging, Italian National Council for Research (IBB-CNR), Area di Ricerca site and Headquarters, Via Pietro Castellino 111, 80131 Naples, Italy*
¶ these authors contributed equally to this work

A

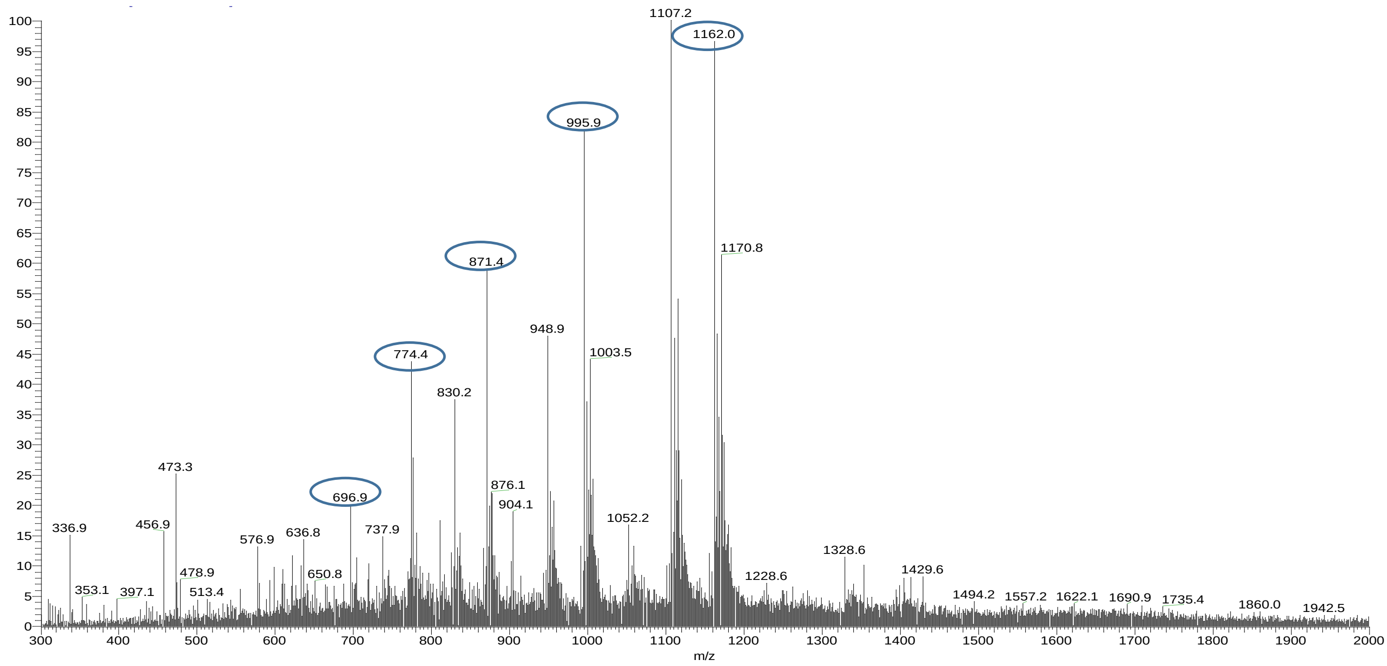

B

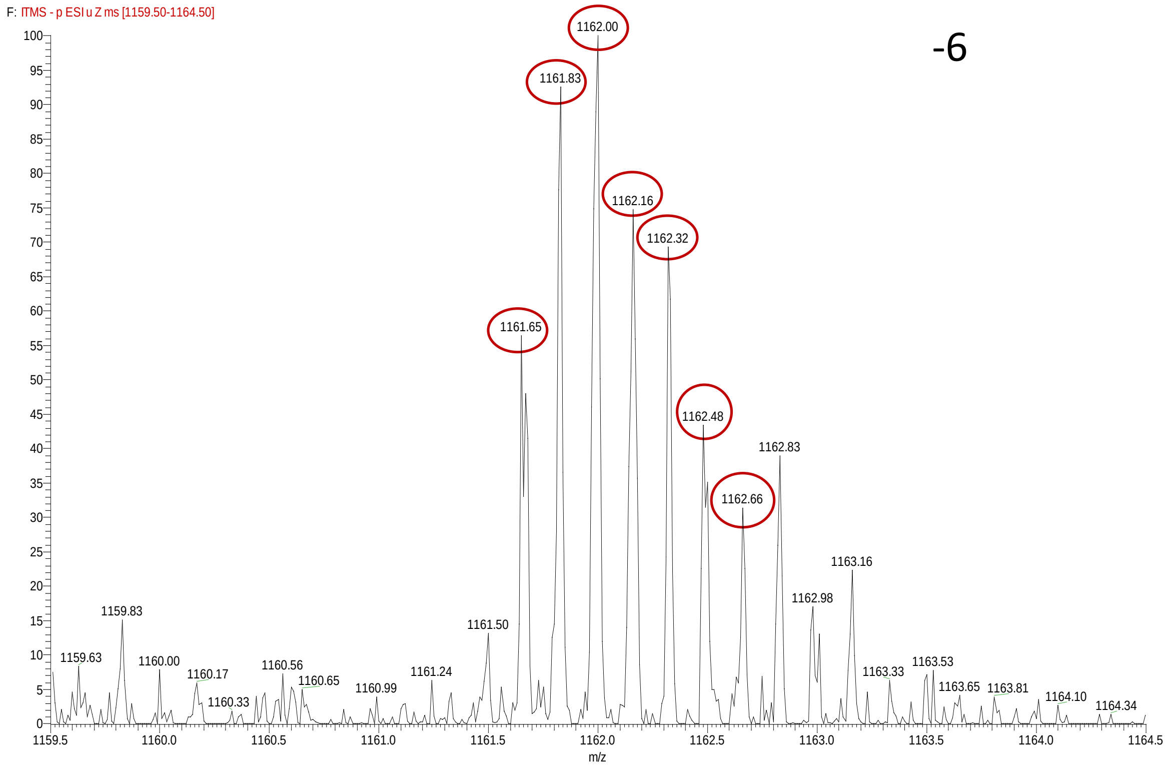

Figure S1 (A,B) Electrospray Ionization Mass Spectrometry (ESI MS) characterization of the d[(GGGGCC)$_3$GGGG] oligomer employed in this study.

Table S1 Interpretation of ESI MS Multi-Charge Ions for the peak corresponding to the d[(GGGGCC)$_3$GGGG] oligomer.

Species Label	Charge Number	MW	Mass per Charge Unit	Found Value
M	-	6940.5	-	-
[M+K - 7H]$^{6-}$	-6	6982.0863	1163.6811	1162.0
[M+K - 8H]$^{7-}$	-7	6980.9858	997.2694	995.9
[M+K - 9H]$^{8-}$	-8	6979.8854	872.4857	871.4
[M+K - 10H]$^{9-}$	-9	6978.7849	775.4205	774.4
[M+K - 11H]$^{10-}$	-10	6977.6844	698.7684	696.9

A

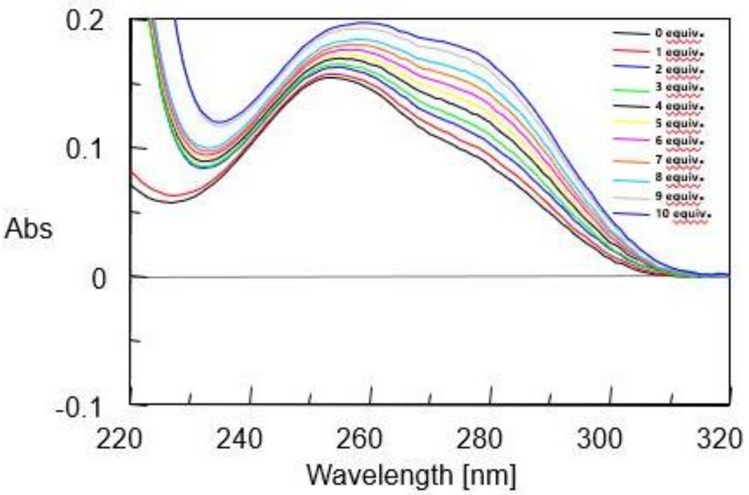

B

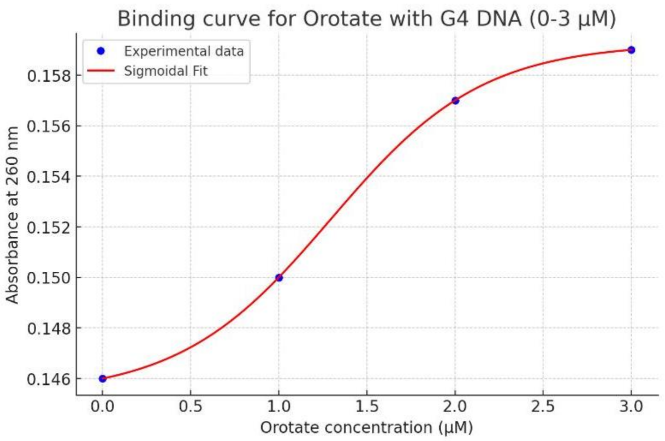

C

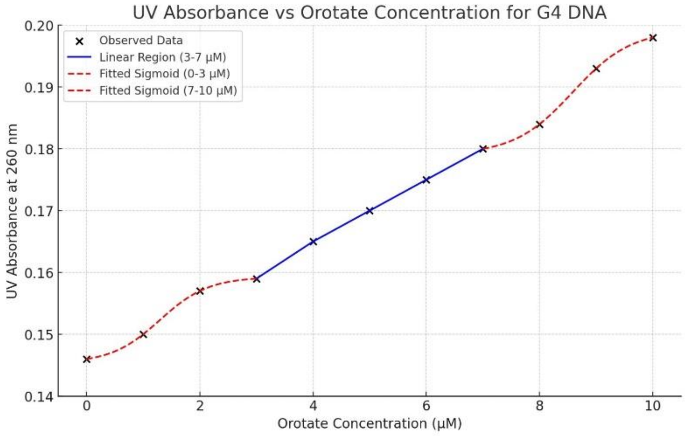

Figure S2 A: UV spectra of orotate solutions in the 10 mM KH_2PO_4 buffer containing 100 mM KCl at 7.0 pH. Concentrations range from 1 to 10 μM. B, C: Binding curve of G4 DNA with orotate, showing UV absorbance at 260 nm versus orotate concentration (0-3 μM, B and 0-10 μM, C). The half-maximum absorbance (related to the binding constant) was estimated from the slope of the sigmoidal curve shown in the figure S2 B, with the concentration at half-max absorbance approximately 1.3 μM

A

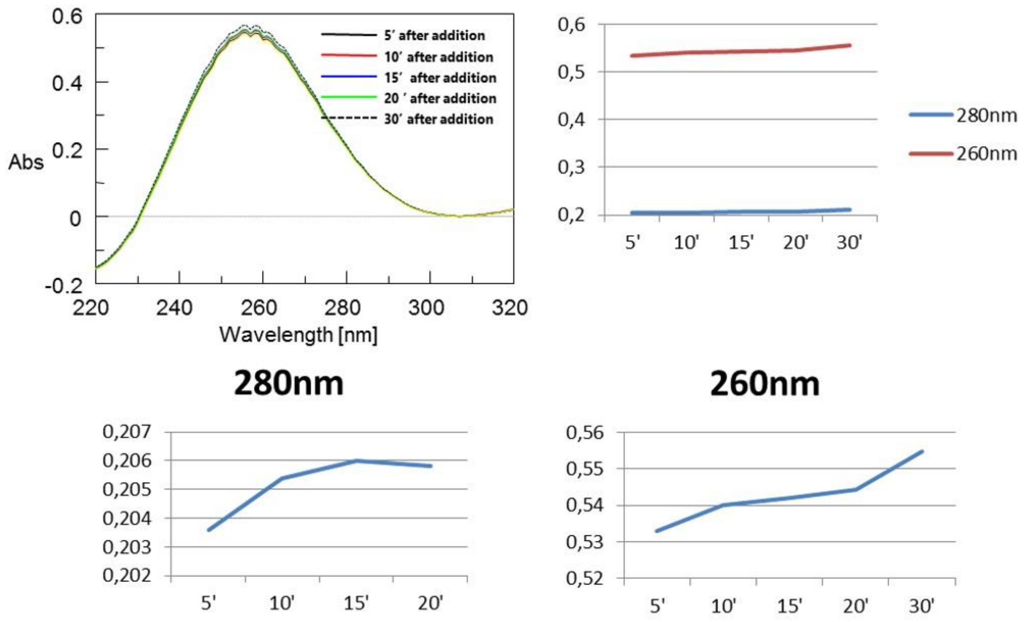

B

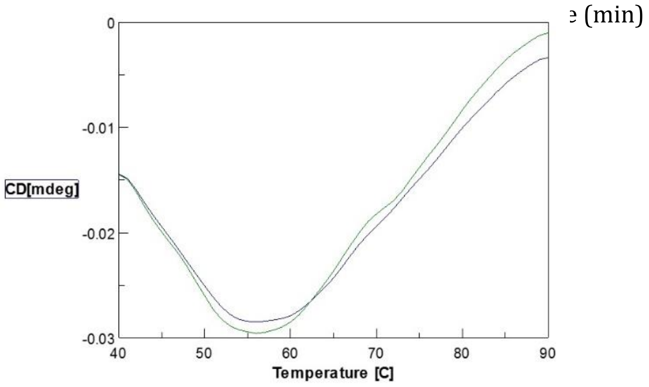

Figure S3 A: UV spectra of the complex formed by orotate and the d[(GGGGCC)$_3$GGGG] DNA at different times.B: Overlapped first derivatives of circular dichroism (CD) spectra of Tel22 (d[AGGGTTAGGGTTAGGGTTAGGG]) DNA in phosphate-buffered saline (PBS, 1×, pH 7.4) at varying temperatures in the absence (blue) and presence (green) of an excess orotate ligand. The CD spectra reveal no shift in the minimum, which is observed at 56°C in both conditions, indicating that orotate does not stabilize the Tel22 G-quadruplex structure under these experimental conditions.

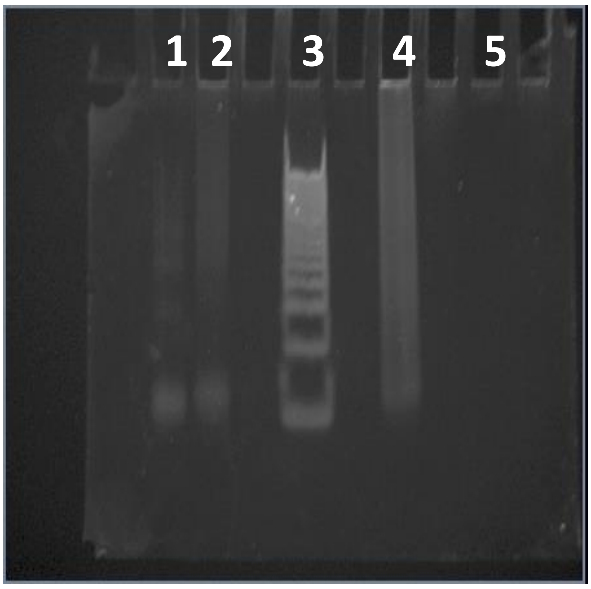

Lane	sample
1	$(GGGGCC)_3GGGG$ + 10 eq. orotate
2	$(GGGGCC)_3GGGG$ + 10 eq. orotate
3	10–100 DNA ladder
4	$(GGGGCC)_3GGGG$
5	Orotate

Fig S4. Gel electrophoresis image stained with Sybr Green, showing the migration pattern of d[$(GGGGCC)_3GGGG$] DNA samples run on a 18% polyacrylamide gel in 1x Tris-Borate-EDTA (TBE) buffer at pH 7.0. The image represents the uncut gel, capturing the distribution of DNA fragments based on their size and charge DNA at different times.

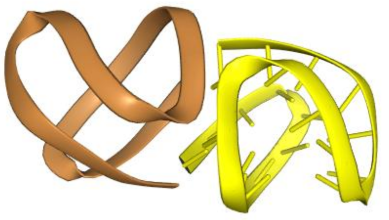

HDOCK score: -212 (highly favoured)

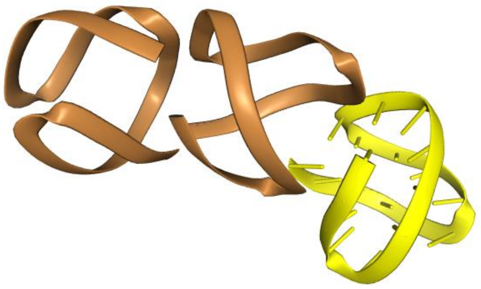

-117 (favoured)

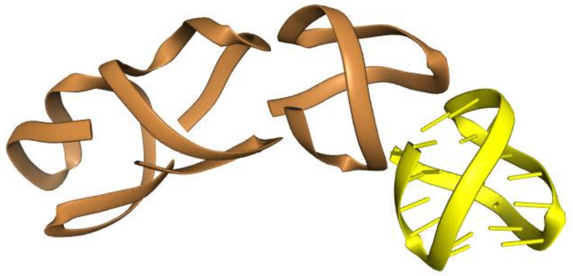

-115 (favoured)

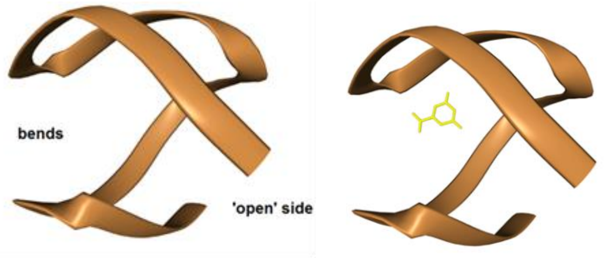

-82score (favoured)

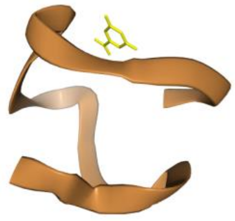

-95.24 (d[TGAGGGTGGGTAGGGTGGGTAA Pu22: parallel model; PDB ID: 1XAV)

Figure S5: This image depicts various views of the d[(GGGGCC)$_3$GGGG] G-quadruplex structure in both its monomer and oligomerized forms and the most stable molecular docking pose illustrating the interaction between the monomeric d[(GGGGCC)$_3$GGGG] G-quadruplex and orotate. The quadruplex structure is highlighted with annotations indicating two distinct regions: the 'bends' and the 'open' side. Notice how the tetramer of the G_4C_2-containing DNA was used as a model of the G_4C_2-containing DNA aggregate for the docking with orotate. It was obtained after repeated 'self-docking' steps starting from the monomer the G_4C_2-containing DNA. The dimerization occurs in a symmetrical manner involving two flanking open sides from two monomers (open/open interaction). This corresponds to a very stable complex formation with a score of -212. The dimer was docked with another the G_4C_2-containing DNA monomer giving a tetramer whose formation in silico involves a bends/open interaction between the dimer and the upcoming monomer and is still energetically favoured (score –117) but less than the previous step. The tetramer was obtained in analogy to the above procedure starting from the trimer and another the G_4C_2-containing DNA monomer. Again, a bends/open interaction is predicted with a score of –115. In other terms, the aggregation involves an initial dimer with higher formation tendency thanks to an open/open interaction. Subsequent oligomerization involves still favourable (but less stabilizing) effects. Note that although the complex between orotate and the parallel G4 DNA is predicted to be slightly stronger (–95.24 vs. –82.22, see bottom of this figure), the parallel form of DNA does not occur experimentally with our sequence, which predominantly adopts the antiparallel conformation.

Molecule 1

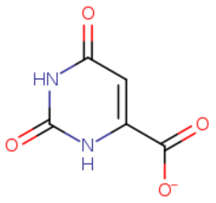

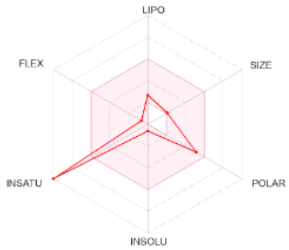

SMILES O=c1[nH]c(=O)[nH]c(c1)C(=O)[O-]

Physicochemical Properties	
Formula	C5H3N2O4-
Molecular weight	155.09 g/mol
Num. heavy atoms	11
Num. arom. heavy atoms	6
Fraction Csp3	0.00
Num. rotatable bonds	1
Num. H-bond acceptors	4
Num. H-bond donors	2
Molar Refractivity	32.70
TPSA	105.85 Å²

Lipophilicity	
Log $P_{o/w}$ (iLOGP)	0.08
Log $P_{o/w}$ (XLOGP3)	-0.83
Log $P_{o/w}$ (WLOGP)	-2.57
Log $P_{o/w}$ (MLOGP)	-1.04
Log $P_{o/w}$ (SILICOS-IT)	0.65
Consensus Log $P_{o/w}$	-0.74

Water Solubility	
Log *S* (ESOL)	-0.62
Solubility	3.75e+01 mg/ml ; 2.42e-01 mol/l
Class	Very soluble
Log *S* (Ali)	-0.91
Solubility	1.90e+01 mg/ml ; 1.22e-01 mol/l
Class	Very soluble
Log *S* (SILICOS-IT)	-0.83
Solubility	2.32e+01 mg/ml ; 1.49e-01 mol/l
Class	Soluble

Pharmacokinetics	
GI absorption	Low
BBB permeant	No
P-gp substrate	No
CYP1A2 inhibitor	No
CYP2C19 inhibitor	No
CYP2C9 inhibitor	No
CYP2D6 inhibitor	No
CYP3A4 inhibitor	No
Log K_p (skin permeation)	-7.84 cm/s

Druglikeness	
Lipinski	Yes; 0 violation
Ghose	No; 4 violations: MW<160, WLOGP<-0.4, MR<40, #atoms<20
Veber	Yes
Egan	Yes
Muegge	No; 1 violation: MW<200
Bioavailability Score	0.56

Medicinal Chemistry	
PAINS	0 alert
Brenk	0 alert
Leadlikeness	No; 1 violation: MW<250
Synthetic accessibility	1.82

Molecule 2

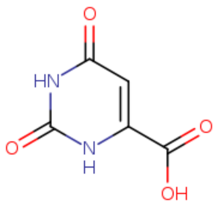

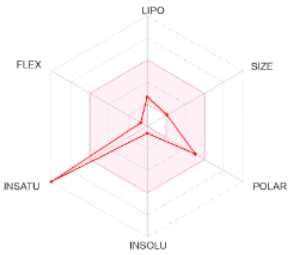

SMILES O=c1[nH]c(=O)[nH]c(c1)C(=O)O

Physicochemical Properties	
Formula	C5H4N2O4
Molecular weight	156.10 g/mol
Num. heavy atoms	11
Num. arom. heavy atoms	6
Fraction Csp3	0.00
Num. rotatable bonds	1
Num. H-bond acceptors	4
Num. H-bond donors	3
Molar Refractivity	34.64
TPSA	103.02 Å²

Lipophilicity	
Log $P_{o/w}$ (iLOGP)	0.08
Log $P_{o/w}$ (XLOGP3)	-0.83
Log $P_{o/w}$ (WLOGP)	-1.24
Log $P_{o/w}$ (MLOGP)	-1.04
Log $P_{o/w}$ (SILICOS-IT)	0.65
Consensus Log $P_{o/w}$	-0.47

Water Solubility	
Log *S* (ESOL)	-0.62
Solubility	3.72e+01 mg/ml ; 2.38e-01 mol/l
Class	Very soluble
Log *S* (Ali)	-0.85
Solubility	2.19e+01 mg/ml ; 1.40e-01 mol/l
Class	Very soluble
Log *S* (SILICOS-IT)	-0.83
Solubility	2.33e+01 mg/ml ; 1.49e-01 mol/l
Class	Soluble

Pharmacokinetics	
GI absorption	High
BBB permeant	No
P-gp substrate	No
CYP1A2 inhibitor	No
CYP2C19 inhibitor	No
CYP2C9 inhibitor	No
CYP2D6 inhibitor	No
CYP3A4 inhibitor	No
Log K_p (skin permeation)	-7.84 cm/s

Druglikeness	
Lipinski	Yes; 0 violation
Ghose	No; 4 violations: MW<160, WLOGP<-0.4, MR<40, #atoms<20
Veber	Yes
Egan	Yes
Muegge	No; 1 violation: MW<200
Bioavailability Score	0.56

Medicinal Chemistry	
PAINS	0 alert
Brenk	0 alert
Leadlikeness	No; 1 violation: MW<250
Synthetic accessibility	1.85

Figure S6 Pharmacokinetic and physicochemical properties assessment of orotate (top) and orotic acid (bottom) using SWISS ADME http://www.swissadme.ch/index.php (accessed on 12 November 2024). Notably, the PAINS (Pan Assay Interference Compounds) score is 0 for both orotate and orotic acid, indicating no predicted off-target alerts and suggesting a low likelihood of non-specific interactions in biological assays.
